# Electromagnetic navigational bronchoscopy‐guided dye marking to identify the subsegmental bronchus in thoracoscopic anatomic subsegmentectomy

**DOI:** 10.1111/1759-7714.14116

**Published:** 2021-08-18

**Authors:** Rongjian Xu, Min Zhao, Yandong Zhao, Yunpeng Xuan, Yi Qin, Wenjie Jiao

**Affiliations:** ^1^ Department of Thoracic Surgery The Affiliated Hospital of Qingdao University Qingdao China; ^2^ Center of Laboratory Medicine Qilu Hospital of Shandong University (Qingdao) Qingdao China

**Keywords:** ENB, subsegmental bronchus, thoracoscopic subsegmentectomy

## Abstract

Video‐assisted thoracoscopic surgery (VATS) subsegmentectomy has been widely used to resect small‐sized lung lesions in clinical practice. Precise identification of the subsegmental bronchus is one of the essential steps in performing thoracoscopic anatomic subsegmentectomy. Here, we report a thoracoscopic right S^2^a segmentectomy with preoperative electromagnetic navigational bronchoscopy (ENB)‐guided injection of methylene blue to identify the subsegmental bronchus in a 51‐year‐old male. We successfully performed complicated surgery using this method. This ENB‐guided dye marking method may accurately distinguish the subsegmental bronchus to effectively guide surgery.

## Graphical abstract

We successfully performed a thoracoscopic right S2a segmentectomy with preoperative electromagnetic navigational bronchoscopy (ENB)‐guided injection of methylene blue to identify the subsegmental bronchus. This ENB‐guided dye marking method may accurately distinguish the subsegmental bronchus to effectively guide surgery.

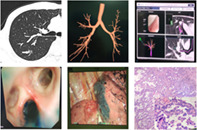

## INTRODUCTION

In recent years, the early detection rate of lung cancer has increased each year in China. Thoracoscopic segmentectomy and subsegmentectomy are performed to treat small‐sized lung lesions, and the validity of these techniques has been established.^1^, ^2^ Based on the complex structure and anatomical variation, accurate identification of the objective bronchus is vital to achieve segmentectomy and subsegmentectomy. In previous studies, many methods have been used to distinguish the objective bronchus and intersegmental plane.^3^, ^4^ However, each method has its limitations, and there is still no way to directly mark the objective bronchus. Therefore, it is necessary to identify a novel and simple method. Here, we describe a modified ENB‐guided dye marking method to identify the objective bronchus. To our knowledge, this is the first publication to describe direct recognition of the subsegmental bronchus using the ENB‐guided dye marking method.

## CASE REPORT

**FIGURE 1:**
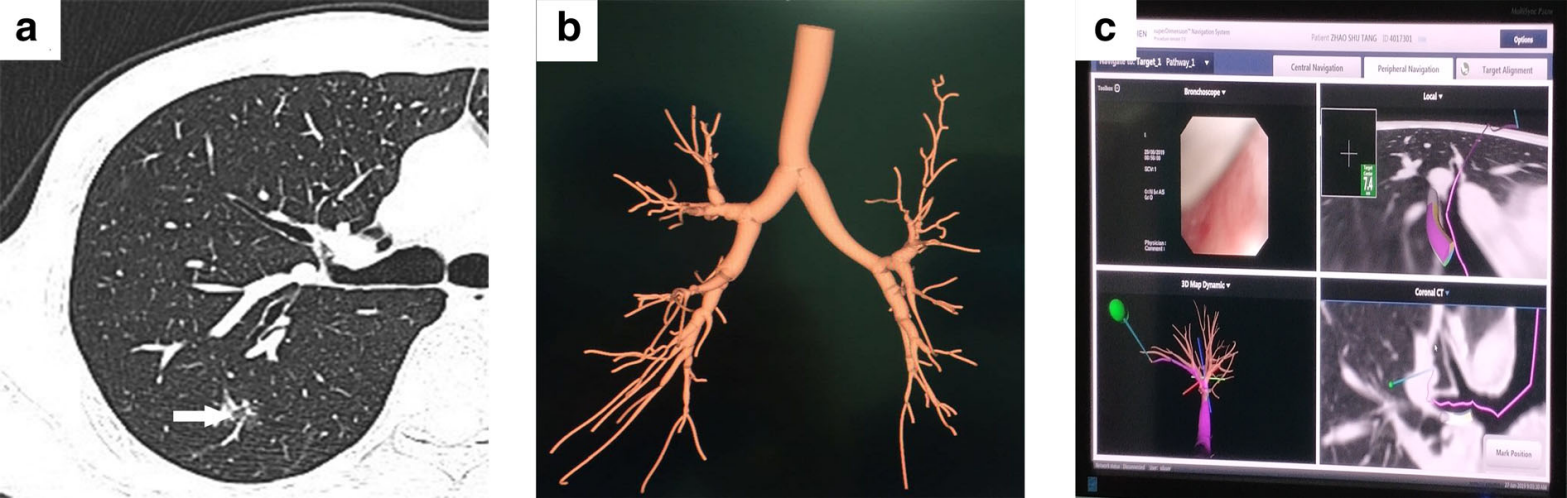
(a) Chest computed tomography (CT) showed a ground‐glass opacity (GGO) (white arrow) located in the right S^2^a subsegment. (b) A 3‐dimensional reconstructed depiction of the bronchial tree using electromagnetic navigational bronchoscopy (ENB) software. (c) Shows the real‐time navigation screen when the ENB‐guided dye marking method was performed

**FIGURE 2:**
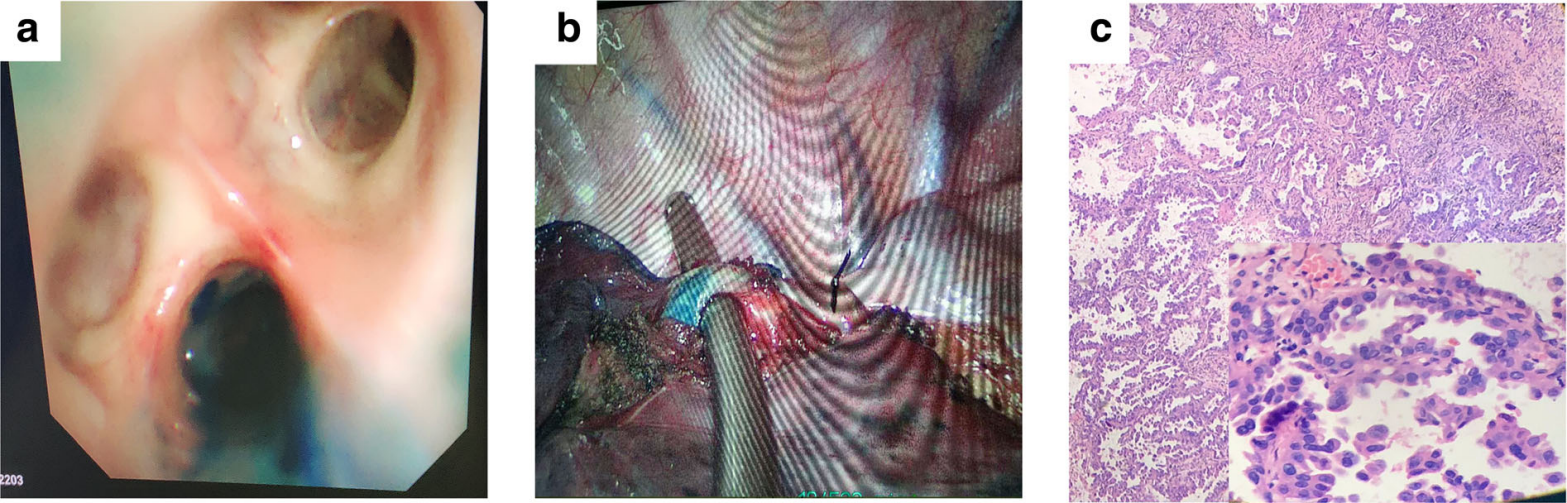
(a) Methylene blue was injected through the bronchial opening under the guidance of electromagnetic navigational bronchoscopy (ENB). (b) The right S^2^a subsegmental bronchus was directly identified during the operation. (c) The pathological result was adenocarcinoma

A 51‐year‐old male presented to our hospital after a pulmonary shadow had been detected 20 months prior (Figures 1 and 2). The patient denied cough, hemoptysis and other symptoms related to thoracic disease. He was previously healthy and had no smoking history. At this time, no measures were taken, but the patient was asked to attend follow‐up. He underwent re‐examination, and chest computed tomography (CT) showed a small ground‐glass opacity (GGO) approximately 0.7 × 0.8 cm in size in the posterior segment of the right upper lobe. The GGO had a rough border and an uneven density (Figure 1a). The patient underwent 2 weeks of anti‐inflammatory treatment. CT was performed to confirm the therapeutic effect. However, no significant change was observed. Routine preoperative examinations were all normal. The patient requested that surgery was performed to achieve a clear diagnosis. After a multidisciplinary discussion, we concluded that the lesion was a single, small‐sized, and pure GGN. The lesion was located in the peripheral part of the lung subsegment. Thoracoscopic anatomical subsegmentectomy could completely resect the tumor. Thus, we decided to perform right S^2^a segmentectomy. The ENB‐guided dye marking method was selected to directly identify the subsegmental bronchus.

ENB (superDimension, Covidien) was performed under general anesthesia. A bronchoscope (Olympus), together with a navigational system, was used as described previously.^5^ The ENB system created an electromagnetic field around the patient's chest. The locatable guide (LG) was inserted into the bronchoscope with the extended working channel (EWC). Once the LG probe was placed within the electromagnetic field, its position in the X, Y, and Z planes, as well as its orientation (roll, pitch, and yaw movements), were captured using the ENB system. This information was displayed on a monitor in real‐time. The monitor displayed a graphic depiction of the position of the sensor probe superimposed on constructed three‐dimensional (3D) CT images of the patient's anatomy in coronal, sagittal, and axial planes (Figures 1b,c). The sensor probe and EWC were successfully steered to the lesion under the guidance of 3D CT images in the “tip‐view” orientation. The closest distance between the sensor probe and the targeted lesion was approximated 0.8 cm. Once the targeted lesion was reached, the sensor probe and EWC were gradually retracted back to the bronchial opening of the S^2^a subsegment. Then, the LG was carefully withdrawn to ensure the EWC was left in situ. Approximately 1.0 ml of methylene blue was injected into the B^2^a through the bronchial opening (Figure 2a). Subsequently, approximately 2 ml of air was injected which took around 15 min to complete. Next, the patient was placed in the left lateral decubitus position, and thoracoscopic right S^2^a subsegmentectomy was performed. The right B^2^a was successfully dye‐marked during surgery (Figure 2b). The pathological diagnosis was adenocarcinoma in situ (Figure 2c). The patient fully recovered and was discharged within 1 week.

## DISCUSSION

In recent years, the early diagnosis rate of lung cancer has increased each year in China. Thoracoscopic segmentectomy has gradually become the predominant treatment for small‐sized lung cancer. However, the anatomical structure of the segment is complex and exhibits individual variation; thus, thoracoscopic segmentectomy, especially subsegmentectomy, is a challenging procedure. At present, several methods have been used to identify complicated segmental structures. Up to now, preoperative 3D CT bronchography and angiography (3D‐CTBA) is the most common method. It not only localizes pulmonary nodules, but it can also reveal the 3D structure of targeted segments. Based on this, 3D printing model can be further applied when needed.^3^ However, each method has its limitations, and it is still necessary to identify a feasible method.

As navigational technology to diagnose and localize small lung lesions, ENB has been successfully applied since 2005.^6^ ENB is a minimally invasive method that combines conventional and virtual bronchoscopy with high accuracy and low complication rates. ENB allows the guidance of the probe to reach the fourth order of bronchi or pulmonary lesions beyond the reach of conventional bronchoscopy.^7^ In the past, ENB‐guided localization or biopsy of small lung lesions was mainly performed by physicians. More recently, ENB‐guided dye marking has been proven as a valuable method in thoracic surgery. The success rate of ENB‐guided localization for small lung lesions ranges from 97.2% to 100%.^8^ In this study, we aimed to explore the feasibility of the ENB‐guided dye marking method to identify the subsegmental bronchus in subsegmentectomy. Our research has confirmed that the ENB‐guided dye marking method is feasible and effective for identification of the subsegmental bronchus. However, this method has its own disadvantages. In our opinion, airway injury and lung injury might occur as major complications, which could lead to bleeding in severe cases. Moreover, the injected amount of methylene blue should be appropriate during surgery. Excess methylene blue may cause incorrect marking of other subsegmental bronchi. The equipment and operating process need to be optimized in future studies.

The ENB‐guided dye marking method has specific applications. First, an adequate assessment of lung lesions should be made before surgery. This can be done using chest CT and 3D‐CTBA. The lesion should be a single, small‐sized (<1 cm), and pure GGN located in the peripheral part of the lung subsegment. Thoracoscopic anatomic subsegmentectomy can be used to completely resect the tumor. Second, the clinician should be skilled in thoracoscopic examination and ENB. Surgeons should also be familiar with the anatomical structure of the lung so that the sensor probe and EWC can be accurately sent to the opening of the objective bronchus. Finally, the correct amount of methylene blue should be released to dye mark the subsegmental bronchus.

Identifying the subsegmental bronchus is the main technical difficulty in subsegmentectomy. 3D‐CTBA and 3D printing model are commonly used methods that reveal the 3D structure of targeted segments before surgery. However, they cannot directly mark the targeted bronchus. Our study verified that ENB‐guided dye marking for direct identification of the subsegmental bronchus during surgery was a feasible method. In addition, this method can locate small lung nodules and provide 3D information on targeted segments.

In conclusion, our study demonstrates that the ENB‐guided dye marking method is feasible for identifying the subsegmental bronchus in thoracoscopic subsegmentectomy. To the best of our knowledge, this is the first report to directly identify the subsegmental bronchus using this method. This approach could shorten the surgical duration and may be worthy of further clinical research.

## CONFLICT OF INTEREST

The authors declare that there are no conflicts of interest.
